# Gender, Race, and Ethnic Representation of Incoming Transplant Hepatology Fellows: A 14-Year Analysis of Fellowship Diversity

**DOI:** 10.1016/j.gastha.2023.04.007

**Published:** 2023-04-26

**Authors:** M.R. Mansour, T.D. Meram, A.M. Rida, E.J. Denha, A.L. Michel

**Affiliations:** 1 Oakland University William Beaumont School of Medicine, Rochester, Michigan; 2 Department of Internal Medicine, Henry Ford Hospital, Detroit, Michigan; 3 Department of Internal Medicine, Beaumont Hospital, Royal Oak, Michigan

See editorial on page 873.

Increasing diversity in the field of medicine is imperative to serving the increasingly diverse US population. It is projected that the majority of the US population will not be represented by a single ethnic group by the year 2043, making the United States a first-time majority-minority nation.^1^ A diverse physician workforce that reflects the ever-evolving US population is necessary to provide culturally competent, patient-centered care. The Association of American Medical Colleges defines Under-represented in Medicine (UIM) as “those racial and ethnic populations that are under-represented in the medical profession relative to their numbers in the general population.” Historically, UIM consisted of individuals identifying as Black or African American, Hispanic or Latinx, American Indian or Alaska Native, and Native Hawaiian or other Pacific Islander.^2^ In recent years, efforts have been made to mitigate the diversity gap in medicine by supporting UIM, which has been reinforced with an upward trend in the percentage of UIM medical school matriculants annually and therefore overall training physicians.^3^ However, particularly in the fields of gastroenterology and hepatology, racial and ethnic diversity has not been extensively explored or addressed.^3^ Minority populations comprise approximately 35% of the US population; yet, only 9% of gastroenterology fellows and 10% of practicing gastroenterologists identify as UIM.^4^, ^5^, ^6^ The purpose of this study was to analyze trends regarding the genders and ethnicities of incoming transplant hepatology fellowship trainees in the United States from the years 2007 to 2021.

Data regarding the self-identified gender and ethnic identities of incoming transplant hepatology fellows from the years 2007–2021 were collected. The publicly available data were extracted from the Graduate Medical Education Census published in the *Journal of the American Medical Association*.^7^ Individual metrics were subsequently analyzed to determine any trends over the 14-year period.

Within this 14-year interval, male trainees comprised 54.2% and female trainees comprised 45.8% of incoming transplant hepatology fellows (Table). Self-identified race and ethnic representation was distributed in decreasing order as follows: White (n = 176, 44.8%), Asian (n = 166, 42.2%), Other/Unknown (n = 37, 9.4%), Black (n = 24, 6.1%), Multiracial (n = 3, 0.8%), Native Hawaiian/Pacific Islander (n = 1, 0.3%), and American Indian/Alaska Native (n = 1, 0.3%) (Table). Of note, data for the Multiracial group were not available for the 2007–2014 years. Thirty trainees (7.6%) identified as Hispanic Ethnicity in addition to one of the aforementioned races (Table). The average yearly rate of change was −0.93% for males and +0.93% for females. Additionally, the average yearly rate of change per the various racial and ethnic backgrounds was as follows: Black +2.04%, White +0.12%, Hispanic Ethnicity −0.35%, Asian −0.76%, and Other/unknown −1.40%. American Indian/Alaska Native, Native Hawaiian/Pacific Islander, and Multiracial groups demonstrated no rate of change, 0% per group. The average yearly rate of change per gender and various racial and ethnic backgrounds is demonstrated in Figure.

**Table:** Number of Incoming Transplant Hepatology Fellows in the Years 2007–2021, Grouped by Race, Ethnicity, and Gender

Year	Black	American Indian/ Alaska native	White	Asian	Native Hawaiian/Pacific Islander	Multiracial	Other/unknown	Hispanic ethnicity	Total	Male	Female
2020–2021	3	0	14	19	0	0	8	2	**44**	20	24
2019–2020	2	0	18	26	0	2	3	2	**51**	27	24
2018–2019	3	0	13	17	0	1	5	4	**39**	24	15
2017–2018	2	0	17	14	0	0	4	1	**37**	18	19
2016–2017	2	0	13	20	0	0	1	1	**36**	20	16
2015–2016	1	0	19	14	0	0	1	1	**35**	20	15
2014–2015	5	0	13	12	1	0	4	2	**35**	18	17
2013–2014	2	0	14	12	0	0	3	3	**31**	18	13
2012–2013	1	1	16	12	0	0	2	2	**32**	18	14
2011–2012	0	0	15	5	0	0	1	4	**21**	13	8
2010–2011	2	0	8	4	0	0	2	3	**16**	8	8
2009–2010	0	0	8	6	0	0	1	4	**15**	11	4
2008–2009	0	0	7	4	0	0	2	1	**13**	5	8
2007–2008	1	0	1	1	0	0	0	0	**3**	1	2
Total	**24**	**1**	**176**	**166**	**1**	**3**	**37**	**30**	**408**	**221**	**187**
%	**6.1**	**0.3**	**44.8**	**42.2**	**0.3**	**0.8**	**9.4**	**7.6**		**54.2**	**45.8**

Number of Incoming Transplant Hepatology Fellows in the Years 2007–2021. Fellows are grouped by Race, Ethnicity, and Gender indicated in bold.

**Figure:**
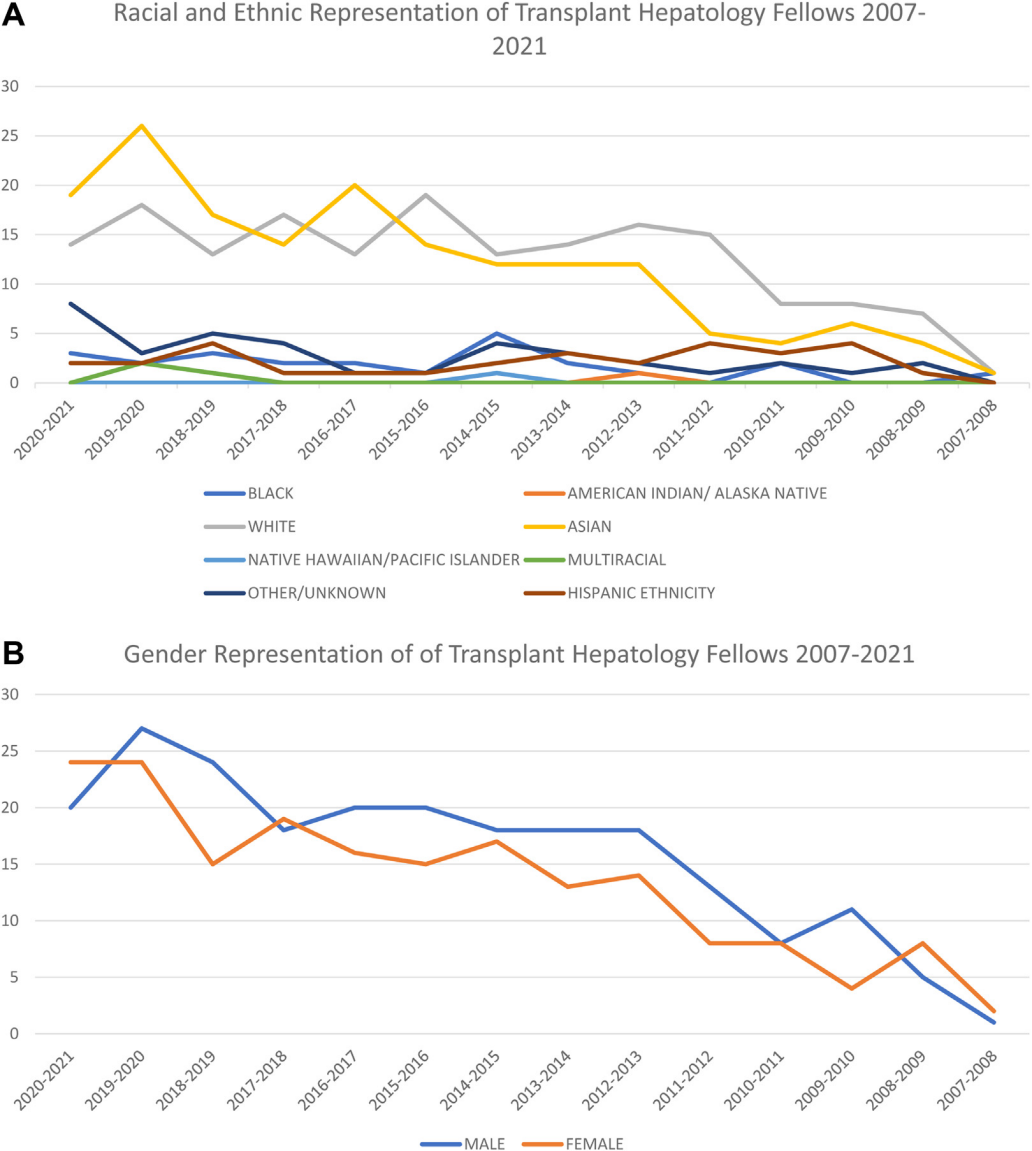
(A) Average yearly rate of change of incoming transplant hepatology fellows in the years 2007–2021, per racial and ethnic group. (B) Average yearly rate of change of incoming transplant hepatology fellows in the years 2007–2021, per gender.

From 2007–2021, the proportion of incoming female transplant hepatology fellows fell among the proportion of female internal medicine residents and females of the general population, estimated at 43.1% and 50.5% in 2021, respectively.^8^^,^^9^ Of note, female representation has slightly increased with a rate of change of 0.93% over the 14-year period. Data analysis also revealed a promising increase in the proportion of incoming Black fellows within this time frame, peaking at 7.7% of matriculants in 2018–2019. This peak, however, is still almost half of the number of Black Americans in the general population.^9^ Interestingly, those of Asian descent represent 6.1% of the general population but constituted 40.7% of matriculants within this time frame.^9^ However, representation of Asian fellows slightly decreased over the years, with the lowest proportion in 2011–2012 still constituting 23.8% of matriculants. This was contrasted by the slight decrease in representation of Hispanic ethnicity over the years, where the proportion of fellows was consistently less than that of the Hispanic group in the overall population.^9^

Undoubtedly, the current cultural climate has prompted recognition of physicians of all gender, racial, and ethnic backgrounds in the fields of gastroenterology and hepatology; however, there are still advancements to be made.^10^ The paucity of diversity in the field is multifactorial. Endeavors to improve proportionate gender, racial, and ethnic representation begin with identifying and breaking down perceived barriers of minority applicants. Barriers may include lack of mentors and sponsors of similar backgrounds as well as limited exposure to available resources.^3^, ^4^, ^5^^,^^10^ Institution-wide education on cultural sensitivity and creation of leadership positions and committees that support UIM while monitoring their progress and success could be prosperous in mitigating the diversity gap. Training the next generation of physicians with a focus on diversity, equity, and inclusion is essential to best serve the needs of the changing US population.

The limitations of this retrospective analysis include a lack of available data to extract for the “Multiracial” group for the academic years 2007–2014 and a small sample size. Additionally, there should be consideration of the various racial and ethnic identities that may not have been included in the original fellowship application and therefore not indexed. Also, the notion that one’s perception of race or ethnicity may vary from one person to another, and therefore trainees of similar backgrounds may be self-categorized in different groups. Data also only regarded the matriculating fellows, which hinder us from identifying if the lack of diversity stems from the pool of applicants within gastroenterology residencies or from those who matched. Despite these limitations, our study highlights the need to further emphasize diversity within transplant hepatology fellowship programs to create a workforce that may best reflect and serve the evolving US population. The authors did not receive financial support for the research, authorship, or publication of this article. Future research would be beneficial to investigate avenues for increasing diversity among trainees.
